# Cognitive training–related volumetric changes in the A36 perirhinal area are associated with mnemonic discrimination gains in older adults: a 7T MRI study

**DOI:** 10.1002/alz70861_108566

**Published:** 2025-12-23

**Authors:** Panagiotis Iliopoulos, Helena M. Gellersen, Boyan Rong, Radoslaw Martin Cichy, Anne Maass, Emrah Düzel

**Affiliations:** ^1^ Institute of Cognitive Neurology and Dementia Research (IKND), Otto von Guericke University, Magdeburg Germany; ^2^ German Center for Neurodegenerative Diseases (DZNE), Magdeburg Germany; ^3^ Department of Education and Psychology Freie Universität Berlin, Berlin Germany; ^4^ Institute of Cognitive Neurology and Dementia Research (IKND), Otto‐von‐Guericke University, Magdeburg Germany; ^5^ Institute of Cognitive Neuroscience, University College London (UCL), London UK

## Abstract

**Background:**

Mnemonic discrimination (MD), the ability to distinguish between similar but distinct memories, relies heavily on the integrity of key medial temporal lobe (MTL) regions. Aging is associated with declines in MD, but evidence suggests that cognitive training may help mitigate this decline. While prior research has shown that such training can improve MD performance, little is known about the relationship between these behavioral improvements and structural changes in specific brain regions. This study investigates whether an 8‐week web‐based MD training program enhances MD in older adults and examines whether these gains are linked to volumetric changes in MTL areas, including the A36 perirhinal area, a critical region for object and scene memory.

**Method:**

A total of 151 older adults (age M = 69.71 years, SD = 4.17) participated in an 8‐week web‐based MD training program. Participants were divided into three groups: object stimuli training (OG), scene stimuli training (SG), and an active control group (AC). A subset of OG (n = 33) and AC (n = 28) participants underwent pre‐ and post‐training 7T MRI scans (MP2RAGE 0.6 mm; T2w imaging: 0.4 x 0.4 x 1 mm along the hippocampal axis). The training required participants to differentiate between similar objects and scenes ('lures') and repeated items ('repeats’) in a progressively challenging MD task. Pre‐ and post‐training, participants completed a behavioral battery assessing MD and cognitive transfer effects. Volumetric changes in key MTL subregions, including the A36 perirhinal area, were assessed using the T2w ASHS algorithm for the MTL areas’ segmentations.

**Result:**

Behavioral results showed significant gains in MD in the OG and SG groups compared to the control group. Notably, within the object training but not the control group, volumetric increases in the left A36 perirhinal area were specifically linked to MD performance gains for scenes (ie. near transfer to scenes) but not objects. This suggests a robust link between structural plasticity and MD behavioral improvement.

**Conclusion:**

Our findings indicate that an 8‐week MD training program enhances memory performance in older adults and induces structural changes in the A36 perirhinal area, highlighting the potential for cognitive training to support brain health during aging.

